# *Scoary2*: rapid association of phenotypic multi-omics data with microbial pan-genomes

**DOI:** 10.1186/s13059-024-03233-7

**Published:** 2024-04-11

**Authors:** Thomas Roder, Grégory Pimentel, Pascal Fuchsmann, Mireille Tena Stern, Ueli von Ah, Guy Vergères, Stephan Peischl, Ola Brynildsrud, Rémy Bruggmann, Cornelia Bär

**Affiliations:** 1 Interfaculty Bioinformatics Unit and Swiss Institute of Bioinformatics, University of Bern, Bern, CH-3012 Switzerland; 2 Graduate School for Cellular and Biomedical Sciences, University of Bern, CH-3012 Bern, Switzerland; 3 Methods development and analytics, Agroscope, Schwarzenburgstrasse 161, Bern, CH-3003 Switzerland; 4 Food microbial systems, Agroscope, Schwarzenburgstrasse 161, Bern, CH-3003 Switzerland; 5 Norwegian Institute of Public Health, Oslo and Norwegian University of Life Science, Ås, Norway

**Keywords:** Prokaryote, Bacteria, Pan-genome, Metabolite, Microbial genome-wide association studies, GWAS, BGWA, Genotype-phenotype association, Fermented food, Omics

## Abstract

Unraveling bacterial gene function drives progress in various areas, such as food production, pharmacology, and ecology. While omics technologies capture high-dimensional phenotypic data, linking them to genomic data is challenging, leaving 40–60% of bacterial genes undescribed. To address this bottleneck, we introduce *Scoary2*, an ultra-fast microbial genome-wide association studies (mGWAS) software. With its data exploration app and improved performance, *Scoary2* is the first tool to enable the study of large phenotypic datasets using mGWAS. As proof of concept, we explore the metabolome of yogurts, each produced with a different *Propionibacterium reichii* strain and discover two genes affecting carnitine metabolism.

**Supplementary Information:**

The online version contains supplementary material available at 10.1186/s13059-024-03233-7.

## Background

The emergence of large-scale whole-genome sequencing, coupled with rapid development of tools for analyzing and sharing data, presents unprecedented opportunities to understand microbial genomics, to establish connections between genetic variations and functions, both at the level of individual organisms and within complex microbial communities. Metabolic models can be used to gain deep insights into bacterial physiology, which is one possible approach to address these questions. However, useful models are challenging to develop, make strong assumptions, and are inherently limited to established and curated networks of genes and metabolites. As the functions of many bacterial genes (around 40–60% [1]) are not yet known, a more straightforward approach is necessary to capture bacterial phenotypes and relate them to genomic data. While this may not lead to a holistic understanding of the microbes, it enables the previously unknown gene functions in an annotation-independent manner.

Concomitantly, recent developments in omics technologies such as mass spectrometry (MS) make it possible to capture massive phenotypic profiles [2], potentially enabling the discovery of novel gene functions in high throughput. Even though sequencing and omics technologies are advancing rapidly, linking these data to gain an understanding of functional relationships remains a major challenge. Numerous and conceptually different approaches have been developed to integrate omics datasets, with a strong focus on human genetics and disease-related tasks such as disease subtyping or biomarker prediction [3]. Among these, only a few attempt to directly link phenotypes measured by omics technologies to genes using established human genome-wide association (hGWAS) or quantitative trait loci (QTL) methods [4]. Unfortunately, due to the differences between human and microbial genomes, hGWAS methods cannot be directly applied to microbes.

Microbial genome-wide association studies (mGWAS), sometimes termed bacterial genome-wide association (BGWA), are still a new area of research with the goal of finding genetic explanations to bacterial phenotypes [5]. The reason why the well-established methods of hGWAS cannot simply be adapted lies in the plasticity of bacterial genomes. In human, the genetic diversity is very low. Therefore, hGWAS is typically performed by aligning reads to a human reference genome and focuses almost exclusively on single nucleotide polymorphisms (SNPs), which amount to more than 99.9% of human genomic variants [6]. Moreover, humans reproduce sexually, and the genome is diploid. Because of recombination, genetic variants that are in proximity have a higher chance of being co-inherited, a phenomenon termed “linkage disequilibrium” that can lead to false positives in GWAS. In contrast, bacteria reproduce clonally, and thus the entire genome is in linkage disequilibrium and population structure becomes a strong confounding factor (pseudoreplication) [7, 8]. Furthermore, microbial genomes are much more diverse. For instance, the core 97% genome of 10,667 *E. coli* genomes represents only 1.96% of the total pangenome [9]. Microbial genomes have varying numbers of circular or linear DNA molecules, sometimes with plasmids or phages, and recombination and mutation rates that may vary considerably between and even within species. Recombination occurs in many species through the processes of transformation, transduction or conjugation [10, 11]. Thus, the focus in mGWAS is often on gene-presence-absence, copy-number-variants, unitigs or k-mers.

A good overview of existing mGWAS software can be found in San et al. [5]. Among the tools presented, Scoary was the most-cited software (as of February 2023), undoubtedly in part due to its simplicity and user-friendliness. Scoary scores binary genomic features (i.e., presence/absence of orthogenes, SNPs, unitigs or *k*-mers) for associations to a binary phenotype using Fisher’s test and accounts for population structure using a post hoc label-switching permutation test. This post hoc permutation test is based on the pairwise comparisons algorithm [12, 13]. A major advantage of this permutation test is that users do not need to experiment with ill-informed mutation rate parameters or inform the program about population structure [14].

Unfortunately, Scoary was neither designed to handle large numbers of microbes nor large phenotypic datasets. Like all mGWAS software we know of, it was created with the purpose of analyzing single phenotypes, often related to pathogenicity and to drug resistance. Three limitations prevent Scoary from such high-throughput analyses: performance constraints, the inability to pre-process numeric traits, and a lack of post-GWAS methods optimized for the substantially expanded output generated in such scenarios.

Here, we present Scoary2, a complete re-write and extension of the original Scoary software, developed to efficiently link phenotypic multi-omics data of yogurt to microbial genomes using mGWAS and enable integrative data exploration of yogurt metabolomes. Scoary2 is significantly faster and can thus be applied to more traits as well as isolates. Moreover, the pre-processing (binning) of continuous phenotypes is now integrated and the types of genomic input-data permitted are expanded. Crucial for efficient post-GWAS data exploration of large datasets, Scoary2 includes a simple frontend implemented in HTML/JavaScript that visually and interactively integrates the data as well as optional metadata describing isolates, traits, and orthogenes. These improvements are also beneficial in ordinary mGWAS use cases. As noted by San et al. [5], many mGWAS solutions are limited in that they lack data pre-processing functionality as well as post-GWAS methods.

We demonstrate Scoary2 using a dataset of bacterial strains belonging to 20 different (sub-)species that were selected from the strain collection of Agroscope, the Swiss center of excellence for agricultural research. The aim of this study was to investigate the effect of the pan-genome of the added bacterial strains on the phenotype of the yogurts.

## Results

### The Scoary2 software

#### Overview

Scoary2 retains all features that are already familiar to users of original Scoary [14]. As in Scoary, the two basic inputs are (i) a table that describes the genotypes (orthogenes, SNPs, *k*-mers, unitigs) present in all isolates and (ii) a table containing the trait(s) of the isolates. These function as explanatory and response variables, respectively. Like in original Scoary, for each trait, Scoary2 generates a list of significant genotypes per trait as output.

Furthermore, the scope and user-friendliness of Scoary have been significantly improved by enhancements and optimizations in Scoary2. The main improvement of Scoary2 is the addition of an interactive data exploration app which greatly facilitates the exploration of the output. To this end, metadata files describing the genotypes, traits, and isolates can be added as input. Moreover, unlike original Scoary, Scoary2 can perform multiple testing correction for all *p*-values that are generated using Fisher’s test and not just per phenotype. Finally, Scoary2 is significantly faster than the original Scoary software.

A manual [15] as well as a tutorial [16] detailing how to use Scoary2 are available on GitHub. Below, we describe the improvements over original Scoary in detail.

#### Performance enhancements

The original Scoary software only had one software dependency (SciPy [17]) and the entire software was implemented using Python-native data structures (i.e., lists and dictionaries) only. In general, Scoary2 uses the efficient NumPy [18] and pandas [19] libraries to load and process the data. Most importantly, the pairwise comparison algorithm was reimplemented, drastically reducing the number of phylogenetic tree traversals. Gene-presence-absence and trait-presence-absence data are now represented as Boolean NumPy arrays, enabling just-in-time compilation of the pairwise comparison algorithm using Numba [20]. To determine the time complexity of this most time-consuming step and compare the new implementation to the original one, we applied both algorithms to randomly generated datasets with varying numbers of genes and genomes. The results are visualized in Additional file 1: Fig. S1. Using symbolic regression, we determined that the most parsimonious formula for computing the runtime is given by this equation: ${\text{runtime}}=\mathrm{constant }\times {n}_{\text{genes}} \times {n}_{\text{genomes}}$. The new implementation is around 30x faster than original Scoary. In addition, confidence intervals in the permutation test only depend on the topology of the gene and the number of isolates with the trait. In a dataset with many traits, the same confidence intervals may be used many times. Thus, caching confidence intervals in an SQLite database [21] reduces the number of times this expensive algorithm is executed. The modular software design makes it possible to import the pairwise comparison from the Scoary2 Python module and re-use the algorithm in different programs. Another substantial speed boost comes from enabling true multiprocessing during binarization and analysis of traits using the producer/consumer software architecture pattern. Also, Scoary2 uses a just-in-time-compiled implementation of Fisher’s test (available as a standalone Python library [22, 23]) which is orders of magnitudes faster than the reference implementation in SciPy. Moreover, original Scoary is limited to analyzing datasets with less than 3000 isolates due to Python’s recursion limit. By dynamically adjusting this limit, Scoary2 can now analyze datasets with up to 13,000 isolates.

Using equivalent settings, Scoary2 is about 59 times faster (23 sec vs 22 min) at analyzing 100 randomly selected traits from the dataset described in this paper (44 isolates, 9051 genes). Scoary2 takes only 16 minutes to process our full dataset (3889 traits, 182 isolates, 10,358 hierarchical orthogroups) with the parameters *n_cpus* = 8, *multiple_testing* = bonferroni:0.1, *n_permut* = 1000, *max_genes* = 50, *trait-wise-correction* = True. The reduced dataset presented in this paper (3889 traits, 44 isolates, 1466 hierarchical orthogroups) takes only 43 s to process with the parameters *n_cpus* = 8, *multiple_testing* = bonferroni:0.999, *n_permut* = 1000, *trait-wise-correction* = True. All measurements were performed on a laptop with an Intel i7-1355U CPU (10 cores, 1.70-5 GHz).

#### Software distribution

Scoary2 can be installed using the python package manager (pip) or used through an official docker container, where all dependencies are bundled, guaranteeing easy installation far into the future, thus ensuring reproducibility.

#### Binning of continuous phenotypes

**Fig. 1:**
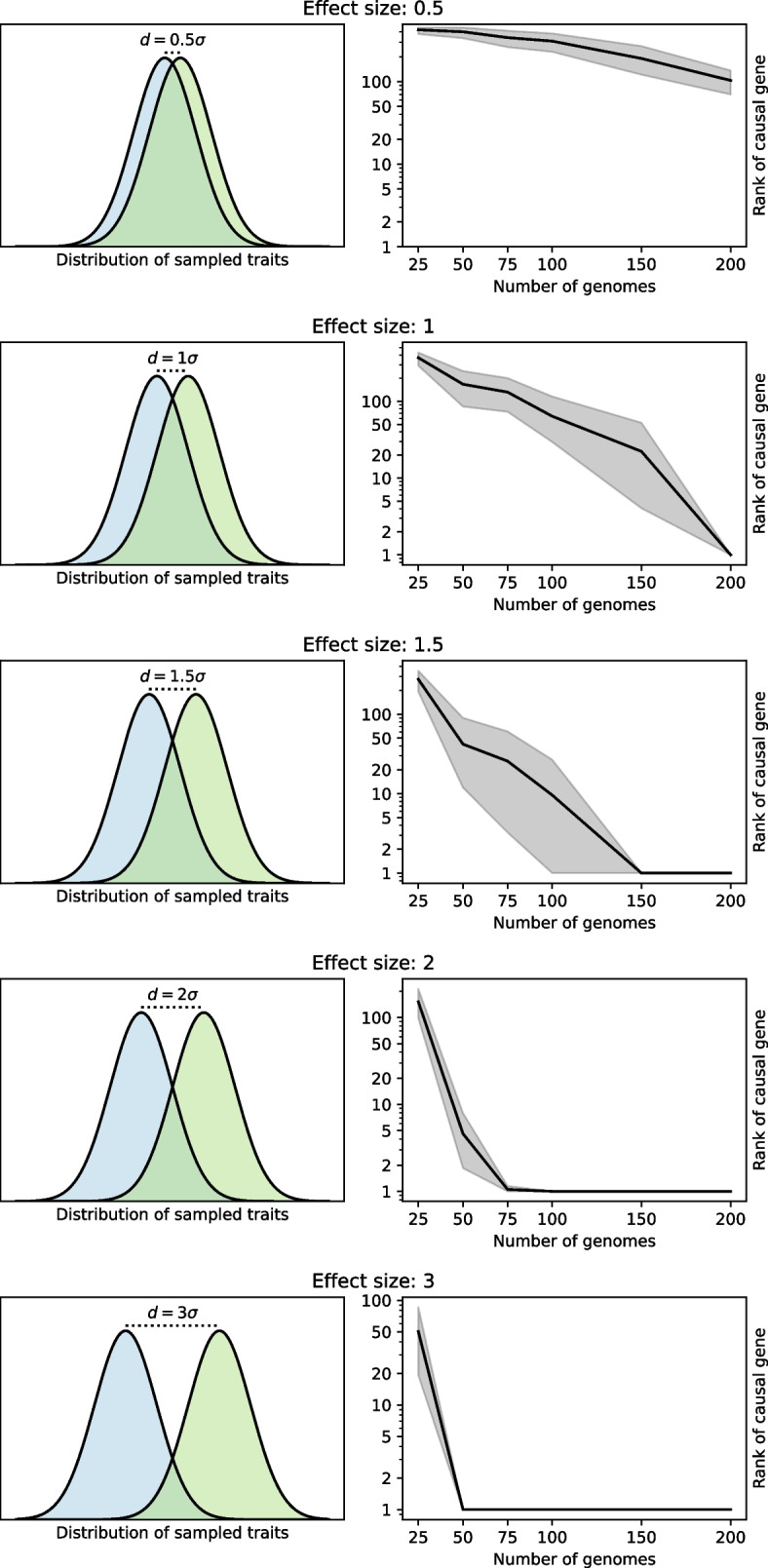
Benchmarking of Scoary2’s automatic binarization based on simulated datasets for different effect sizes. Panels on the left show the distributions from which the numeric phenotype was sampled based on the presence or absence of a designated causal gene. Panels on the right indicate the rank of the causal gene in the output of Scoary in relation to the number of genomes in the simulated dataset. The black line indicates the average rank and the grey area indicates 90 % confidence interval based on 20 simulations

The core algorithm of Scoary is based on binary genotype and phenotype data. Scoary2 is newly capable of automatically pre-processing continuous phenotypes into binary ones. This enhancement is essential for datasets with so many traits that they cannot be curated manually, though this is otherwise recommended. For this purpose, two Scikit-learn [24] methods, *k*-means and Gaussian mixture model, are available. The former will classify all isolates as having or lacking the trait. The Gaussian mixture model seeks to fit two Gaussian distributions and calculates the probability of each isolate having or not having the trait. By default, isolates that are classified with less than 85% predicted posterior probability are ignored from further analysis. The fitting of Gaussian mixture models can fail, and the user can decide whether to skip such traits or use k-means as a backup instead. In the data exploration app, the original continuous values are used again to calculate a histogram. To assess the power of the automatic binarization, we simulated genomic datasets of varying sizes, see Fig. 1 and the “Methods” section for details.

#### OrthoFinder support

The name Scoary was chosen in homage to the orthology inference software Roary [25], which transformed bacterial comparative genomics in 2015 thanks to its speed and user-friendliness [26]. However, Roary does not seem to be under active development anymore and was not included in recent *Quest for Orthologs* benchmark studies [27]. Today, OrthoFinder is the most accurate ortholog inference method according to this benchmark [27, 28]. It is under continued development and is among the most used tools in the field. As input, original Scoary uses Roary’s *gene-count* table, which indicates how many genes per orthogroup each genome has. However, this makes it cumbersome to find the relevant genes of an interesting orthogroup. While Scoary2 is still compatible with the *gene-count* table, it is highly recommended to use the *gene-list* table, produced by both Roary and OrthoFinder, where cells contain a list of gene identifiers. This way, the gene names will be shown in the data exploration app.

#### Output and data exploration app

Scoary2 produces similar tables as output as original Scoary. As San et al. [5] indicated, the ability to add annotations to orthogroups would “contribute immensely” to the utility of mGWAS tools. For datasets with many phenotypes, this is absolutely essential. Therefore, Scoary2 does not just allow to add metadata to orthogroups but also to traits and isolates. In addition, Scoary2 contains a simple data exploration app for easy inspection of the results. It was built using the JavaScript libraries Bootstrap, Papa Parse, Slim Select, DataTables, Plotly, and Phylocanvas [29–34]. The data exploration app was developed to be available as a standalone software library [35] which could be re-used for other mGWAS tools, further extending its usefulness. It consists of two pages (*overview.html and trait.html*), which are described in next two paragraphs.

**Fig. 2:**
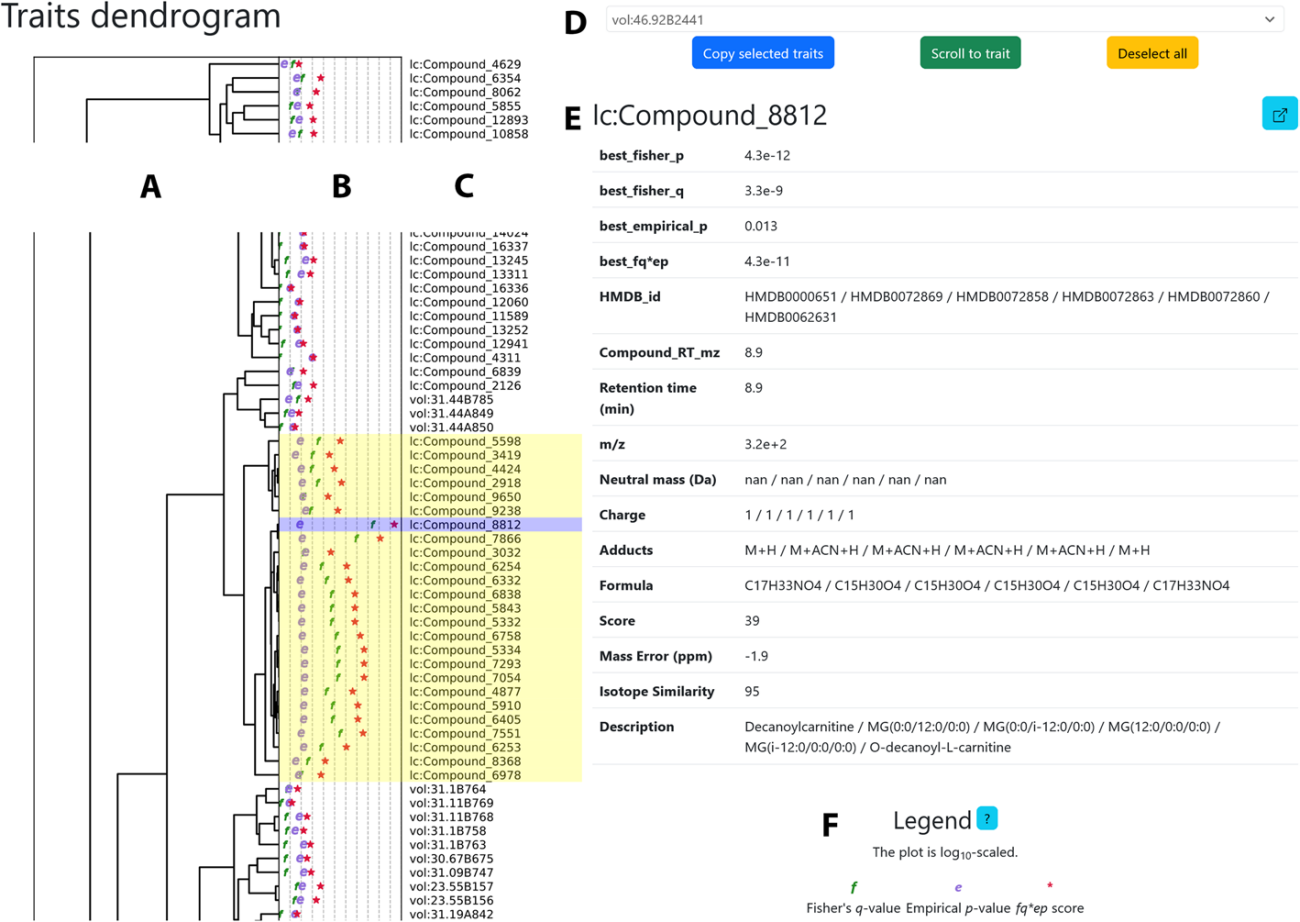
The first page (overview.html) of the Scoary2 data exploration app. **A** Dendrogram of traits. A cluster of carnitine-related traits is highlighted in yellow; the highest-scoring trait is selected (blue). **B** Negative logarithms of the *p*-values calculated by Scoary2: *p*-values range from high (left) to low (right); *f* stands for the *p*-value from Fisher’s test, *e* for the *p*-value from the post hoc test, and * for the product of the two values. **C** Trait names. **D** Trait search and navigation tool. **E** Trait metadata. It is updated when the mouse hovers over the traits in the dendrogram. **F** Plot legend

The first page, *overview.html* (Fig. 2), shows a dendrogram of all traits with a significant association to at least one gene. By default, the dendrogram is calculated using the Pearson correlation coefficient for numeric traits and the Jaccard index for binary traits. The distance metrics are made symmetrical to ensure that highly correlated and highly anti-correlated traits end up close to each other in the dendrogram. The negative logarithms of the corrected *p*-value from Fisher’s test, the *p*-value from the permutation test, and the product of the two values are presented next to the dendrogram. These plots, created with SciPy and matplotlib [17, 36], can show at least 20,000 traits. When the mouse pointer hovers over a trait, the associated metadata is presented.

**Fig. 3:**
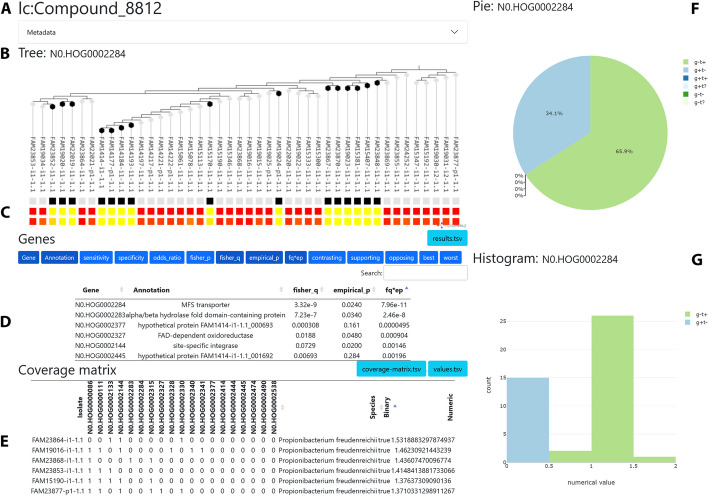
The second page (trait.html) of the Scoary2 data exploration app. **A** Trait name. **B** Phylogenetic tree of the isolates. **C** Top row: presence (black)/absence (white) of orthogene. Middle row: binarized trait. Bottom row: continuous trait. **D** List of best candidate orthogenes with associated *p*-values. **E** Coverage matrix: The numbers in the cells tell the number of genes in the genome that have the annotation. **F** Pie chart that shows how the orthogene and the trait intersect in the dataset. **G** Histogram of the continuous values, colored by whether each isolate has the orthogene (g+/g−) and the trait (t+/t−)

The second page, *trait.html* (Fig. 3), allows users to further investigate each trait. This page includes a phylogenetic tree of the isolates, where color bars indicate which isolates have the trait and which have a selected orthogroup. In addition, a pie chart shows the fraction of isolates that have the trait and how many of these have the gene. If the trait data is continuous, a histogram is also displayed. These plots are updated whenever the user clicks on an orthogroup. Below the phylogenetic tree, there are two tables. The first displays the Scoary statistics and, if present, metadata for each orthogroup. The second table is a *coverage matrix*, which shows the number of genes each isolate has from each orthogroup. If the isolates have metadata, this information is also displayed in this table. If Scoary2 uses an OrthoFinder-style *gene-list* table as input, clicks on these numbers reveal the gene identifiers. Moreover, the data exploration app can be configured to generate hyperlinks, such that clicks on gene identifiers forward the user to a certain URL, for example one where more information about the gene is available, such as its sequence and annotations. Clicks on orthogroups can also be configured to redirect to custom URLs, for example to enable a comparison of the genes.

### Analysis of yogurt dataset

#### Overview of the full dataset

**Fig. 4:**
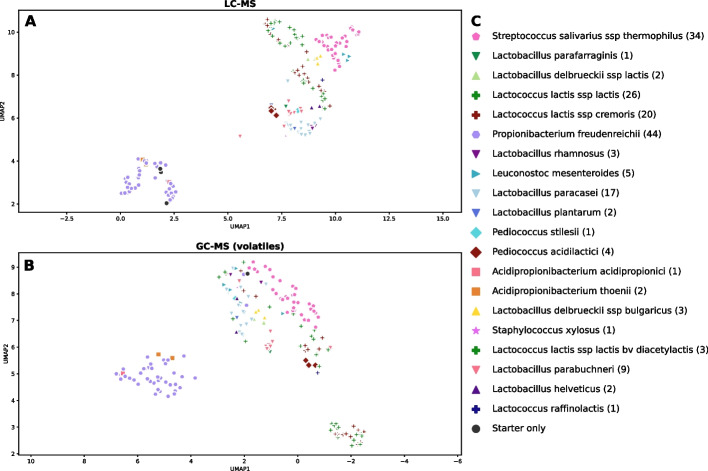
UMAP projections of mass spectrometry datasets. Each symbol represents one yogurt that was made with a different bacterial strain in addition to the starter culture YC-381. **A** LC-MS dataset: 2348 metabolites. **B** GC-MS volatiles dataset: 1541 metabolites. **C** Legend: each (sub-)species has a unique combination of color and symbol. The number in brackets indicates the number of yogurts made using the respective (sub-)species

To illustrate the problem of taxonomy-based clustering of bacterial metabolome data using real data, Fig. 4A/B show 2D embeddings of the full LC-MS and GC-MS volatiles datasets (182 strains) that was generated using uniform manifold approximation and projection (UMAP) [37]. Notably, yogurts made with closely related strains tend to cluster together. Both datasets show one cluster dominated by yogurts made with strains from the order *Propionibacteriaceae* and another dominated by *Lactobacillales*. This means that most correlations between genes and traits are simply due to population structure and to find promising links between genes and traits; mGWAS methods are indispensable.

However, since the analysis of the full dataset would go beyond the scope of this publication and the results can be replicated by restricting the dataset to the 44 *Propionibacterium freudenreichii* isolates, only this selection of data is shown.

#### Scoary2 results

Initially, Scoary2 was employed on the dataset with multiple testing parameters suited to yield robust results (*multiple-testing* = fdr_bh:0.1). Because traits without at least one gene with a significant Fisher’s *q*-value are automatically removed from the output, only 20 metabolites remained [38].

**Fig. 5:**
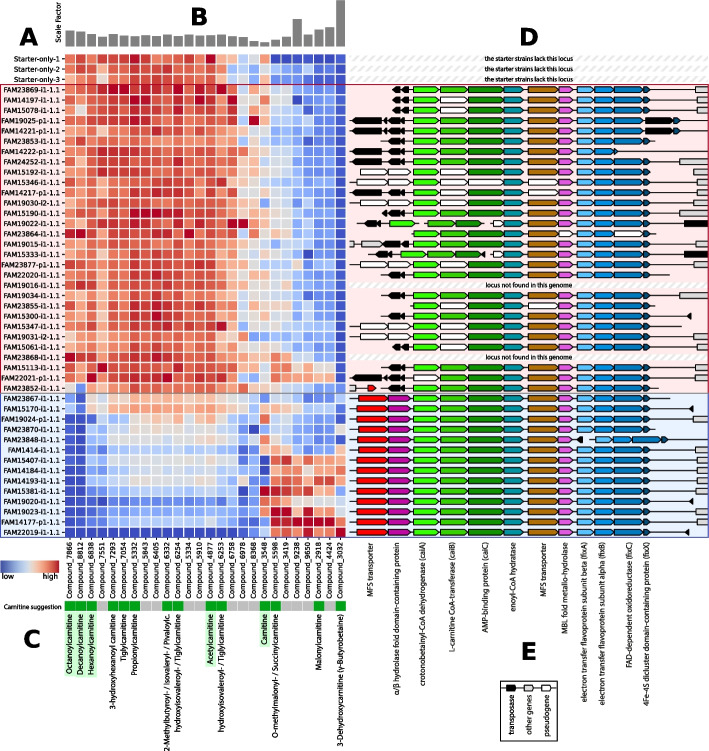
Abundance of the metabolites that correlate with the putative carnitine transporter and corresponding gene loci of three yogurts made from starter cultures only and 44 yogurts made with additional *Propionibacterium freudenreichii* isolates. The figure is divided into two parts, depending on the completeness of the carnitine gene cluster of the isolates: the isolates on a blue background have a complete gene cluster, and the isolates on a red background have an incomplete gene cluster, resulting in varying metabolite compositions. **A** Heat map of the scaled metabolite abundances. Scale: blue (low) to average (white) to red (high). **B** Scale factor of each metabolite. **C** Color bar that indicates whether the mass spectrometry database suggested a match with carnitine in the name (green) or not (grey). The suggested names are shown below. Names highlighted in green were confirmed with standard substances. **D** Comparison of the associated gene cluster spanning from the MFS transporter (red) to *fixX* (dark blue). **E** Annotations of the orthogroups. Genes that belong to the same orthogroup are highlighted in the same color. The *caiABC* genes are colored in shades of green and the *fixABCX* genes in shades of blue. The putative carnitine transporter and hydrolase identified using Scoary2 are highlighted in red and violet, respectively

To explore the relationship of these 20 metabolites within a broader context, we applied Scoary2 again with relaxed multiple testing parameters (*trait-wise-correction* = True, *multiple-testing* = bonferroni:0.999) [39]. This adjustment yielded an output comprising 707 traits, including many false positives. As illustrated in Fig. 2, the original 20 metabolites persisted in the same dendrogram group, indicating strong correlation or anti-correlation. A few additional metabolites were also grouped with the original metabolites, further enriching the contextual understanding. Because each metabolite’s metadata is available in *overview.html* (Fig. 2E), we quickly noticed that the MS database putatively labeled 14 out of 26 of the metabolites in this group as compounds with carnitine in their names (Fig. 5D).

Looking at the results in more detail using *trait.html*, shown in Fig. 3, we found that two genes correlate strongly with these metabolites: an MFS transporter and an α/β-hydrolase fold domain-containing protein. A closer look at the gene identifiers suggests that the two genes are adjacent. Furthermore, the gene loci (Fig. 5E/F) were compared using OpenGenomeBrowser [40] via custom URLs as mentioned earlier, revealing that the two genes are indeed adjacent and located in a syntenic gene cluster, one gene away from an L-carnitine CoA transferase (*caiA*). In the isolates which lack the two genes, many of the clusters were seemingly disjoined by transposases and other genes on the cluster were pseudogenized (Fig. 5E/F).

#### Confirmation of identities for carnitine compounds

The identities of five metabolites (decanoylcarnitine, octanoylcarnitine, hexanoylcarnitine, carnitine and acetylcarnitine), assigned to the gene cluster detected by Scoary2 (Fig. 5D), were subsequently confirmed by LC-MS analysis of pure analytical standard solutions (Table 1).

**Table 1:** List of MFS-transporter-associated metabolites that were confirmed by standard injection

**Metabolite**	**Measured m/z**	**Database match**	**CAS no.**	**Mass error [ppm]**	**Retention time error [%]**
**lc:Compound_8812**	316.24764	Decanoylcarnitine	3992-45-8	< 1	0.67
**lc:Compound_7866**	288.21635	Octanoylcarnitine	25243-95-2	2.02	0.47
**lc:Compound_6838**	260.18515	Hexanoylcarnitine	22671-29-0	< 1	2.13
**lc:Compound_4877**	204.12298	Acetylcarnitine	3040-38-8	< 1	9.66
**lc:Compound_3548**	162.11237	Carnitine	541-15-1	< 1	8.69

Compared to yogurt made from starter cultures only, we found that two thirds of the *Propionibacterium freudenreichii* isolates did not strongly affect the composition of the carnitine-related metabolites shown in Fig. 5. These yogurts are characterized by high amounts of certain acylcarnitines. In contrast, the presence in isolates of the two genes identified by Scoary2 (MFS transporter and α/β-hydrolase fold domain-containing protein) did influence the abundance of those acylcarnitines. Yogurts prepared using such isolates contain depleted amounts of acylcarnitines, particularly octanoylcarnitine and decanoylcarnitine, and are characterized by higher amounts of carnitine, γ-butyrobetaine (putative), and certain other (putative) acylcarnitines.

#### Literature completes the picture

Interestingly, the cluster includes the *caiABC* and *fixABCX* genes, which are associated with the anaerobic metabolism of carnitine [41]. Homologs of *fixABCX* were originally characterized in *Rhizobium meliloti* where they function as a respiratory chain, providing electrons for nitrogen fixation [42]. The genes *caiABC* were first identified as part of the *E. coli caiTABCDE* operon, which is close to and co-expressed with the *fixABCX* operon and together ferment carnitine to γ-butyrobetaine in anaerobic conditions and absence of preferred substrates [41, 43, 44]. This biochemistry is summarized in Additional file 1: Fig. S2. However, the selected *Propionibacterium freudenreichii* isolates are lacking homologs of the crotonobetainyl-CoA hydratase *caiD* and the carnitine/γ-butyrobetaine antiporter *caiT*. Instead, between *caiABC* and *fixABCX*, we find an MFS transporter and an enoyl-CoA hydratase, which might fill these gaps in the pathway. On the other hand, the two genes identified by Scoary2 are also an MFS transporter and a hydrolase, and since only the strains with these genes have a strong impact on the carnitine composition of the yogurt (Fig. 5), it appears that the full operon is required to permit efficient import of precursors and fermentation of carnitine in *Propionibacterium reichii*. This is supported by the apparent degradation of the gene cluster through transposases and pseudogenization in many genomes where the two genes were lost.

## Discussion

### Challenges in linking large phenotypic datasets to genes

#### The need for mGWAS

The taxonomy-based clustering of the metabolomic data (Fig. 4) poses a major problem when trying to find causal connections between orthogenes and metabolites, as the strongest correlations in the dataset are between the many metabolites and orthogenes that also strongly correlate with the population structure. Though these orthogenes may be good predictors of metabolism, most are not causally related to metabolites. To avoid spurious associations in this scenario, and to pinpoint real causal relationships, mGWAS methods such as Scoary’s pairwise comparisons are essential.

#### Automatic binarization

Numeric datasets need to be binarized for the pairwise comparisons algorithm to work. For smaller datasets, this should be done manually and carefully. This is not possible for large phenotypic datasets like the one described in this paper, requiring automatic binarization. We benchmarked our binarization approach using simulated datasets (Fig. 1). The results indicate that with just 50 isolates, Scoary2 is very likely to identify a causal gene with a strong effect (3 σ) as the top-ranked gene. As the effect size decreases, more isolates are required. Interestingly, identifying genes with relatively weak effects (1.5 σ) within the top four ranks with 90 % probability is possible with just 75 isolates.

#### Automated data exploration

We strongly agree with San et al. on the immense utility of post-GWAS methods [5]. To our knowledge, Scoary2’s post-GWAS data exploration app stands out among other mGWAS tools, being able to integrate (i) the detected associations between traits and genes, (ii) relations between traits, (iii) relations between isolates, and (iv) metadata describing traits, genes, and isolates.

While these innovations are very convenient for small datasets, they are an absolute necessity for datasets with many traits. The dendrogram of traits in *overview.html* helps discover groups of (anti-)correlated traits, and the *p*-values plots help to prioritize them. The presence of trait metadata enabled us to notice quickly that many metabolites of one group were annotated as carnitines. Navigating to *traits.html* with only one click allows us to see the phylogeny of the isolates as well as the distribution of the selected trait and the highest-scoring orthogene. The orthogene annotations may also be insightful here. The gene IDs in the *coverage matrix* may reveal that certain orthogenes are often close to each other on the genome, indicating an operon. If the trait is numeric, the histogram may be useful to gauge how strongly the trait varies in the dataset and whether the data points contradicting the hypothesis might just have been incorrectly classified during binarization.

If the app is connected to external comparative genomics tools, it becomes easy to study the candidate gene in more detail. In our example, OpenGenomeBrowser [40] enabled us to discover that the two genes most strongly associated with carnitines are located on the same gene cluster and near an *L-carnitine CoA transferase*, providing more evidence for a causal relationship.

Given that the output from most mGWAS software is structurally similar, i.e., consisting of coefficients for genes and traits, this app offers the possibility to be adapted to other tools. To facilitate this adaptability, the data exploration app exists in its own GitHub repository [35], and we made an effort to design it in a manner that is versatile and not overly specific to Scoary2.

### Potential use in microbial specialized metabolites discovery

Scoary2 may enable a novel discovery strategy for microbial metabolites, thereby providing the potential to accelerate progress in microbiology, drug discovery, and targeted production of functional fermented food to support human health [45, 46]. After all, as outlined in van der Hooft et al. [47], traditional methods are based on established knowledge and labor-intensive experiments, such as activity-guided fractionation of metabolite extracts. These were complemented by genome and metabolome mining approaches. More recently, a “metabologenomic integration” approach was developed that combines high throughput metabolomics with genomics [47]. However, this approach does not take population structure into account and is limited to biosynthetic gene clusters (BGCs), which are challenging to predict, and depends on high-quality genome sequences as well as existing knowledge [48–51]. Scoary2, on the other hand, is conceptually simpler and therefore applicable to a wider range of data, in addition to being easier to use. It is fast enough to process entire metabolomes, cannot just take BGCs but all orthogenes into account, is aware of population structure, and does not rely on existing knowledge and thus represents a valid alternative in that context.

### Comparison with existing mGWAS approaches

The field of mGWAS software is very diverse. Various conceptually different approaches have been developed and refined, and as a result, different tools require different input types and yield conceptually different outputs, and it is unclear how they compare. The main result from LASSO and random forest is the model’s predictive performance. While the parameters of LASSO models are easy to understand, LASSO may randomly choose one of multiple highly correlated genes and drop the others, and random forest does not yield easily interpretable coefficients for the genes. Linear mixed models yield a straightforward *p*-value for each gene, while homoplasy-based methods like treeWAS [52] and Scoary give multiple *p*-values for different types of association scenarios, arguably requiring more careful interpretation. Consequently, tools based on different approaches are difficult to compare. Moreover, benchmarks are often carried out based on simulated datasets, and it is difficult to tell how closely they imitate bacterial evolution and real datasets. We noticed that Scoary and treeWAS were evaluated using simulations that emphasized the evolutionary scenarios they were designed to detect [14, 52], while the simulations from Saber et al. [8], benchmarking linear-model-based tools, did not investigate the effect of homoplastic mutations. We recommend that future research should compare the various approaches using realistic simulations and real datasets and flesh out guidelines as to which approach and tool is recommended in which scenario.

### Limitations of the Scoary2 algorithm

#### Fisher’s test

Fisher’s test is a simple and fast test that measures how strongly a gene and a trait correlate. To determine a *causal* relationship in mGWAS, however, its assumptions are violated, and the resulting *p*-values should rather be interpreted as scores. For users who simply want to learn which traits are associated with specific genes in a tree without any assumptions on causal relation, Fisher’s test is nonetheless useful.

#### Pairwise comparisons

To be as generalizable and widely applicable as possible, the pairwise comparisons algorithm is devoid of any explicitly defined models of evolution and sacrifices some statistical power. For example, a gene whose presence is one hundred percent correlated with a particular phenotype might not be considered significant if the variant-phenotype combination is clustered on a single branch, in other words, if it can be traced back to a single event in the phylogenetic history of the input data. However, we prefer the pairwise comparisons algorithm to explicitly defined models because in our opinion, the mutation rates at every branch in the tree are most often unknown or unavailable. Thus, in Scoary2, only the branching pattern of the phylogenetic tree matters. This means that any errors in its topology could confound results.

A clear downside to the pairwise comparisons algorithm is that it can only deal with binary phenotypic events and not continuous or Brownian motion-type transitions. In Scoary2, phenotypes measured on a continuous scale are automatically binarized with either k-means or a Gaussian mixture model. For the former, there is a risk of improper phenotypic classification, and the latter discards values that do not clearly fit either of the gaussian means, leading to a reduced dataset to draw conclusions on. The latter issue is partially mitigated by manual inspection of the numerical values in *traits.html*.

#### Future directions

In the future, tests that can better exploit numerical data, can detect several types of evolutionary scenarios, or have higher statistical power could be added to Scoary2. Possible candidates are the three tests from treeWAS [52], though there is still room for the development of new algorithms [53]. Alternatively, our data exploration app could be added to the existing tools since it is already a standalone software package.

## Conclusions

We expanded Scoary’s applicability to datasets containing tens of thousands of traits by significantly increasing the performance of the algorithm. Moreover, we added a novel interactive data exploration app that combines trait, genotype, and isolate metadata, greatly facilitating the interpretation of results and crucial for timely exploration of large datasets. We illustrated Scoary2’s capabilities by applying the software to a large MS dataset of yogurts made from different strains of *Propionibacterium freudenreichii*, allowing us to identify novel genes involved in carnitine metabolism. Scoary2 is, to the best of our knowledge, the first software that makes it feasible to study large phenotypic multi-omics datasets using mGWAS. It enables and facilitates the discovery of previously unknown bacterial genotype-phenotype associations and can thus help overcome a major bottleneck in microbial research, namely the unknown role of many genes and their impact on the phenotype. Therefore, it may significantly contribute to fermented food research, accelerating and facilitating the development of fermented food products with specific properties. In addition, Scoary2 has the potential for broader application, for example in basic microbial research, drug discovery and clinical research, and could thus considerably impact microbiological science in the future.

## Methods

### Benchmarking the pairwise comparisons algorithm

Datasets were randomly generated for each combination of [5, 10, 15, …, 100] genes and [5, 10, 15, …, 100] genomes. In addition, a binary trait was randomly generated for each dataset. Both the original Scoary algorithm and the new Scoary2 implementation were applied to each dataset. To ensure robustness in the evaluation, each algorithm was applied to the datasets five times, and the average time taken for computation was recorded. Furthermore, to gain insights into the time complexity of the algorithms, symbolic regression [54] was employed to estimate a parsimonious formula that relates the algorithm’s runtime to the number of genes and genomes in the dataset. The results are visualized in Additional file 1: Fig. S1. The code is available on the Scoary2 GitHub repository [55].

### Benchmarking the runtime of Scoary and Scoary2 on 100 randomly selected traits

One hundred traits were randomly selected from our dataset and binarized. The resulting dataset was then processed with original Scoary using the parameters *permute* = 1000, *correction* = I, *p_value_cutoff* = 0.1 and Scoary2 using the equivalent parameters *multiple_testing* = native:0.1, *n-permut* = 1000, *trait_wise_correction*. The code is available on the Scoary2 GitHub repository [56].

### Benchmarking the automatic binarization

We simulated datasets with 25, 50, 75, 100, 150, and 200 genomes in the same way as described in the original Scoary paper [14]. We generated 20 replicates for each dataset size. We then simulated a numeric phenotype for effect sizes 0.5, 1, 1.5, 2, and 3 as follows: for each genome, if a specified causal gene was not present, a numeric phenotype was sampled from a normal distribution centered on zero and a standard deviation of one. Conversely, if the causal gene was present, the numeric phenotype was sampled from a normal distribution centered on the effect size. We then applied Scoary2 to the dataset and determined the rank of the causal gene in the output. The results are visualized in Fig. 1. The code is available on the Scoary2 GitHub repository [57].

### Yogurt production

Lactose-free, homogenized, pasteurized, semi-skimmed (1.5%) milk purchased from a local retailer was used for yogurt production (Aha! IP Suisse, Migros, Switzerland). Fermentation was carried out overnight (16 h) at 37 °C using the yogurt culture Yoflex® YC-381 (Chr. Hansen, Denmark) containing *Lactobacillus delbrueckii* subsp. *bulgaricus* and *Streptococcus thermophilus* as well as one of the selected strains from the Liebefeld culture collection. The yogurts were stored at − 20 °C until analysis.

### GC-MS (volatiles) dataset

Untargeted volatile analysis was carried out using an Agilent 7890B gas chromatography (GC) system coupled with an Agilent 5977B mass selective detector (MSD) (Agilent Technology, Santa Clara, CA, USA). For volatile analysis, 250 mg of yogurt containing 25 μl ISTD (Paraldehyde 0.5 ppm, Tetradecane 0.25 ppm and D4-Decalactone 0.5 ppm) diluted in water were placed in 20 mL HS vials (Macherey-Nagel), hermetically sealed (blue silicone/Teflon septum (Macherey-Nagel)) and measured in a randomized order. After incubation of the samples for 10 min at 60 °C, the headspace was extracted for 5 min at 60 °C under vacuum (5 mbar) as described by Fuchsmann et al. [58], using the Vacuum transfer in trap extraction method. The trap used was a Tenax TA (2/3 bottom)/Carbosieve S III (1/3 top) (BGB analytics). The temperature of the trap was fixed at 35 °C and the temperature of the syringe at 100 °C. The sorbent and syringe were dried for 20 min under a nitrogen stream of 220–250 mL min^−1^. Desorption of the volatiles took place for 2 min at 300 °C under a nitrogen flow of 100 mL min^−1^. For this purpose, the programmable temperature vaporization injector (PTV) was cooled at 10 °C for 2 min, heated up to 250 °C at a rate of 12 °C sec^−1^ and held for 20 min in solvent vent mode. After 2 min, the purge flow to split vent was set to 100 mL min^−1^. The separation was carried out on a polar column OPTIMA FFAPplus fused silica capillary column 60 m × 0.25 mm × 0.5 μm (Macherey-Nagel) with helium as the carrier gas at a flowrate of 1.5 mL min^−1^ (25.3 cm sec^−1^). The oven temperature was held for 5 min at 40 °C, followed by heating up to 240 °C at a rate of 5 °C min^–1^ with a final holding time of 55 min. The trap was reconditioned after injection at a nitrogen flow of 100 mL min^−1^ for 15 min at 300 °C. The spectra were recorded in SCAN mode at a mass range between m/z 30 to m/z 350 with a gain at 10 with a solvent delay of 4 min. The samples were measured twice in random order. Only compounds that were detected in > 50% of QCs were retained (1541 metabolites).

### LC-MS dataset

Untargeted metabolomic analysis was performed using an UltiMate 3000 HPLC system (Thermo Fisher Scientific) coupled to maXis 4G+ quadrupole time-of-flight mass spectrometer (MS) with electrospray interface (Bruker Daltonik GmbH). Chromatographic separation was conducted on a C18 hybrid silica column (Acquity UPLC HSS T3 1.8 μm 2.1 × 150 mm, Waters, UK), reversed phase at a flow rate of 0.4 mL min ^−1^. The mobile phase consisted in ultrafiltered water (Milli-Q® IQ 7000, Merck, Germany) containing 0.1% formic acid (Fluka™, Honeywell, USA) (A) and acetonitrile (Supelco®, Merck, Germany) with 0.1% formic acid (B), with the following elution gradient (A:B): 95:5 at 0 min to 5:95 at 10 min; 5:95 from 10 to 20 min; 95:5 from 20 to 30 min. The spectra were recorded from m/z 75 to m/z 1500 in positive ion mode. Detailed MS settings were as follows: collision-induced dissociation: 20 to 70 eV, electrospray voltage: 4.5 kV, endplate offset: 500 V, capillary voltage: 3400 V, nitrogen flow: 4 mL min^−1^ at 200 °C, spectra acquisition rate: 1 Hz in profile mode, resolution: 80,000 FWHM. The yogurt samples were measured in triplicates in random order and the values averaged afterwards.

The QC-based robust locally estimated scatterplot smoothing signal correction method was applied for signal drift correction [59] using R (v.3.1.2) [60]. Metabolites with poor repeatability, i.e., detected in < 50% of QCs, were removed, as well as metabolites with a relative standard deviation > 30% in the QC samples. Features that had a median in the QC samples that was < 3 times higher than the median calculated for the blanks were also excluded. This reduced the number of metabolites from 17,310 to 2348.

### Identification of carnitines

The Human Metabolome Database (27) was used with a 5-ppm mass accuracy threshold for the identification of a selection of metabolites. Identity suggestions from databases were then confirmed by MS fragmentation data (when available) and with the injection of pure standards solutions. All standards were purchased at Sigma-Aldrich (Sigma-Aldrich Chemie GmbH, Buchs, Switzerland).

### OrthoFinder

Hierarchical orthogroups were called using OrthoFinder [28] version 2.5.4 with default parameters.

### Locus plots

The gene locus plots (Fig. 5) were generated using OpenGenomeBrowser [40], which utilizes DNA Features Viewer [61], and modified using Adobe Illustrator [62].

### Supplementary Information

**Additional file 1: Fig. S1.** Performance and time complexity of Scoary2’s pairwise comparisons algorithm compared to the original Scoary. **Fig. S2.** Anaerobic Carnitine Reduction in Escherichia coli, adapted from Walt 2002 [44], Bernal 2008 [69] and Ledbetter 2017 [70].

**Additional file 2.** Review history.

## Data Availability

Software The Scoary2 source code is publicly available at https://github.com/MrTomRod/scoary-2/ under the MIT License [63] and from Zenodo (10.5281/zenodo.10352170 [64]. An official docker container (troder/scoary-2) is available on Docker Hub [65]. Datasets The genomes of the 44 *Propionibacterium freudenreichii* used in this study were uploaded to NCBI GenBank and available under BioProject PRJNA946676 [66]. This data is also available via the OpenGenomeBrowser demo instance hosted at https://opengenomebrowser.bioinformatics.unibe.ch/ [67]. The combined LC-MS and GC-MS datasets and the hierarchical orthologs file generated by OrthoFinder are available in the Mendeley Data repository ( 10.17632/yytybr3t4y.1) under the CC BY 4.0 license [68].
