# Supplementary material for: Scoary2: rapid association of phenotypic multi-omics data with microbial pan-genomes
Source: Genome Biol. 2024 Apr 11;25:93. doi: 10.1186/s13059-024-03233-7 (PMC11007987; doi:10.1186/s13059-024-03233-7)
Supplement: Supplementary file 1 — Additional file 1: Fig. S1. Performance and time complexity of Scoary2’s pairwise comparisons algorithm compared to the original Scoary. Fig. S2. Anaerobic Carnitine Reduction in Escherichia coli, adapted from Walt 2002 [44], Bernal 2008 [69] and Ledbetter 2017 [70]. [file 13059_2024_3233_MOESM1_ESM.pdf]

Additional file 1 for "*Scoary2*: rapid association of phenotypic multi-omics data with microbial pan-genomes"

This file includes:

Figures S1 to S2

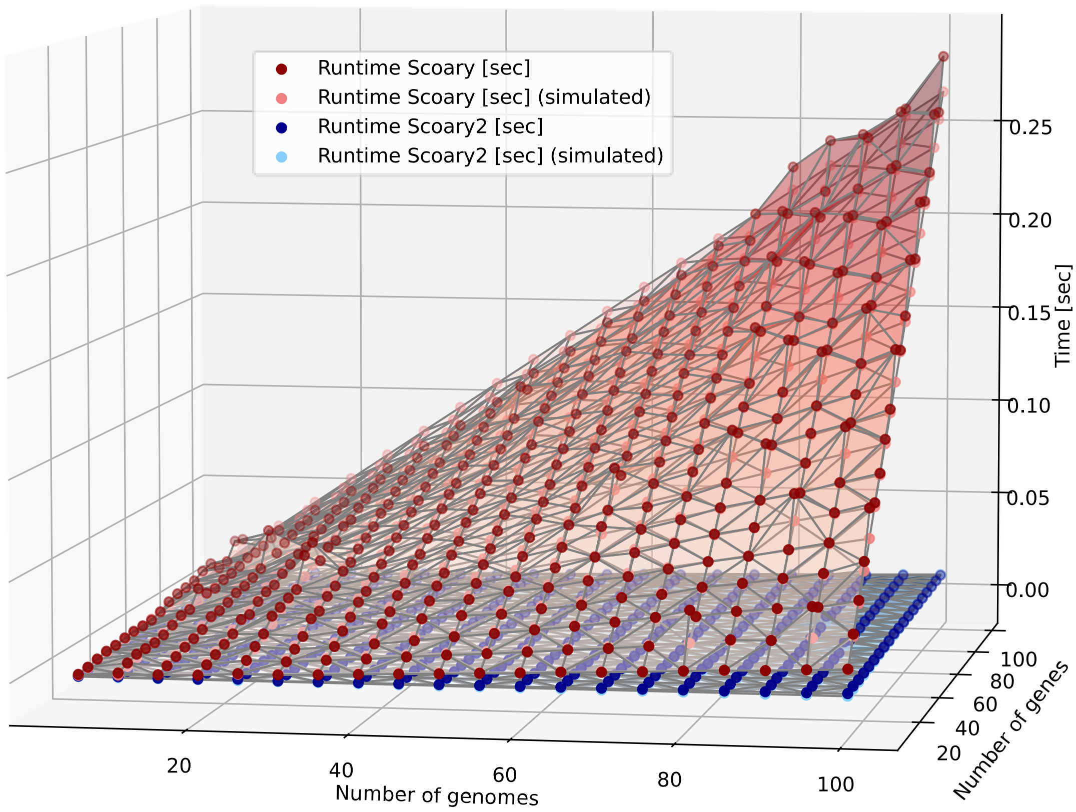

Fig. S1 Performance and time complexity of Scoary2's pairwise comparisons algorithm compared to the original Scoary. Datasets of varying numbers of genomes and numbers of genes were randomly generated and both implementations were applied and timed. Dark red dots represent the runtime of Scoary2, dark blue dots the runtime of original Scoary. Each dot represents the average of five measurements. Using symbolic regression, we discovered that the most parsimonious formula for computing the runtime of both algorithms is given by this equation: $runtime = constant \times n_{\text{genes}} \times n_{\text{genomes}}$. On a laptop with a Intel i7-1355U CPU, the constants were 8.68×10^{-7} for Scoary2 and 2.67×10^{-5} for original Scoary, meaning that Scoary2 is approximately 30x faster. These models are plotted using light red and light blue dots, respectively.

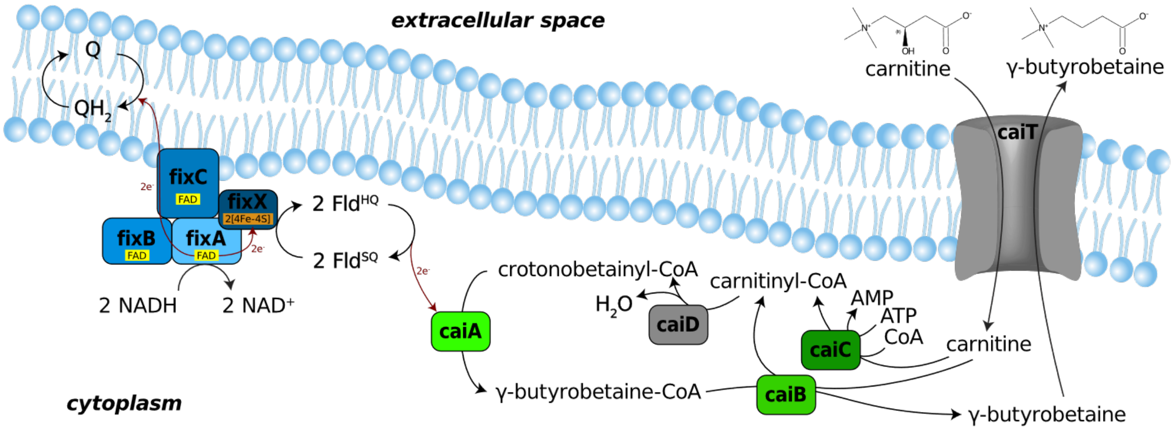

Fig. S2 Anaerobic Carnitine Reduction in *Escherichia coli*, adapted from Walt 2002 [44], Bernal 2008 [68] and Ledbetter 2017 [69]. Proteins that were not found in any of the tested *Propionibacterium freudenreichii* strains are colored grey. ***caiT***: antiport of carnitine and γ-butyrobetaine. ***caiC***: generates initial carnitinyl-CoA. ***caiD***: dehydration of carnitinyl-CoA to crotonobetainyl-CoA. ***caiA***: reduction of crotonobetainyl-CoA to γ-butyrobetainyl-CoA using electrons from *fixABCX*. ***caiB***: recycles CoA moiety. ***fixABCX***: Oxidation of NADH is coupled to reduction of ubiquinone (Q) and flavodoxin semiquinone (Fld^{SQ}), which then delivers electrons to a terminal electron acceptor.

legend: FAD: flavin adenine dinucleotide; [4Fe-4S]: iron-sulfur cluster; Fld^{SQ}: flavodoxin, semiquinone form; Fld^{HQ}: flavodoxin, hydroquinone form; Q: ubiquinone; QH_2: ubiquinol
