# Supplementary material for: Scoary2: rapid association of phenotypic multi-omics data with microbial pan-genomes
Source: Genome Biol. 2024 Apr 11;25:93. doi: 10.1186/s13059-024-03233-7 (PMC11007987; doi:10.1186/s13059-024-03233-7)
Supplement: Supplementary file 2 — Additional file 2. Review history. [file 13059_2024_3233_MOESM2_ESM.docx]

Review History

**First round of review**

**Reviewer 1**

**Were you able to assess all statistics in the manuscript, including the appropriateness of statistical tests used?** There are no statistics in the manuscript.

**Were you able to directly test the methods?** Yes.

**Comments to author:**

This manuscript is essentially a fusion of two quite distinct papers. The first describes updating the popular Scoary software for pangenome GWAS analysis originally published in 2016. The second is analysis of metabolites in experimental yoghurt cultures, which uses the new Scoary2 software, which serves as a proof of concept of using Scoary for high-dimensional analysis combining GWas and metabolite data.

Most changes in Scoary2 are "under the hood" software engineering enhancements that allow the program to run faster and handle larger data sets and new output formats. Another new feature is the ability to binarize continuous variables using a couple of well-known approach. These updates will certainly be welcomed. While the original software was popular for bacterial GWAS, it slowed down considerably when run on more than a few hundred genomes. Nevertheless, the new software is still limited to 13,000 input isolates, which is small compared the current frenetic pace of bacterial genomes sequencing (some species already have 100,000s of public genomes) and limits future its use truly large scale phenotypic testing.

The software was run on yogurts made using 44 different strains of Propionibacterium freudenreichii with a species pangenome of 1,466 hierarchical orthogroups. Thousands of traits - compounds identified by LC-MS were generated for each sample. Exploring the output of SCOARY analysis revealed a cluster of carnitine compounds were correlated in their concentration and were associated two previously unannotated genes near the carnitine biosynthesis cluster. The functions of these genes were not validated experimentally.

This combination is awkward, for example the two paragraphs of the intro doesn't mention the Scoary software, which is advertised by the title as the main theme but instead discusses the genomics of food fermentation. In general, the article spends more time on the results of the yoghurt metabolite study rather than the software. The discussion in particular is over-long and extrapolates the results from the proof-of-concept study without the benefit of any experimental follow-up. Each of the sub-themes is an iterative rather, than highly significant advance on the previous state of the art.

SPECIFIC POINTS

Page 5-6 "Scoary2 results" section.

It is not clear what the 3889 traits measured are - that number is not mentioned in the methods. The section mentions "1,256 metabolites". How are these related to the 3,889 traits? Clarifications needed in this section. Also - were the p-values corrected for multiple hypothesis tests (ie from screening 3,889 traits).

Page 5 line 55. There doesn't seem to be a point to introducing the first dataset that wasn't analyzed in this paper. It illustrates software performance but only anecdotally. A detailed study of performance under different parameters is missing from the study.

Page 6 line 5. I found this para a bit confusing because it talks about clustering of traits between isolates and not GWAS. This could be made clearer.

The figures (especially figure 4) contain too much information and make it hard to parse out the key results. Screenshots of software output should be in the supplement for a research article.

**Reviewer 2**

**Were you able to assess all statistics in the manuscript, including the appropriateness of statistical tests used?** Yes, and I have assessed the statistics in my report.

**Were you able to directly test the methods?** Yes.

**Comments to author:**

Summary

The authors present Scoary2 - a rewrite of the Scoary method for performing microbial GWAS. In addition to rewriting Scoary, the v2 method adds an option to automatically binarize non-binary phenotype data with the use of a Gaussian mixture model or k-means clustering, support for newer orthology inference software, and a data exploration program. The features in Scoary2 are demonstrated on a single dataset consisting of multi-strain yogurt samples.

Since Scoary is undoubtedly widely used for microbial GWAS, a rewrite of the method will no doubt be well received and the data exploration subprogram in particular seems extremely useful to me. If the exploration program can be expanded to support other methods, or supports a format that is easy to extract from other methods, then this would be a very useful development and a major new contribution in the paper. Regarding the new features in Scoary2, however, I think the paper needs some improvement in demonstrating their usefulness which I've outlined in more detail below.

Major issues

Scoary2 provides an option to automatically binarize non-binary phenotypes by clustering the phenotype observations into two clusters. I feel that this sort of automation should be done very carefully and only after consideration by the user, and in general it would be better to explicitly model non-binary features rather than forcing continuous/multi-category data to a binary format. In most cases where binarization is useful, it is (in my opinion) unlikely that the data would easily admit to binary phenotypes found with a simple clustering step. If this feature is retained in Scoary2, I think the paper needs to include a simulation or other benchmark that evaluates the performance of the binarization in a case where the data does not lend itself well to binarization.

The authors claim that other approaches to microbial GWAS such as linear mixed models make "hard-to-verify assumptions about bacterial evolution". It would be useful to explicitly write out what these assumptions are and include a benchmark demonstrating how Scoary2 handles cases where these assumptions do not hold and the other models fail.

The population structure correction using a permutation test, if I understand it correctly, seems to ignore structure in the data that arises from eg. sampling many isolates from the same outbreak/source, or strong lineage structure in the input genomes. In this case the labels should ideally be permuted in a way that considers whole blocks/lineages at a time rather than switching a single label, or otherwise takes the phylogenetic relationships into account.

Scoary2 uses a pairwise comparison algorithm, which presumably scales quadratically in the number of inputs meaning that the method would unlikely scale to tens or hundreds of thousands of isolates. Is there an upper limit to how many isolates Scoary2 can realistically handle, or is it rather limited by the Python recursion depth to 13 000? The presented benchmark only contains 182 isolates, which makes it hard to evaluate the method's scaling.

To place Scoary2 in context with other approaches to microbial GWAS, it would be useful to see Scoary2 applied on a dataset where the genotype-phenotype associations have been extensively studied. One such possibility would be applying Scoary2 to the penicillin resistance data used in this tutorial: https://pyseer.readthedocs.io/en/master/tutorial.html. In this case the genetic determinants are well known (see eg. this review for this particular case https://www.microbiologyresearch.org/content/journal/mgen/10.1099/mgen.0.000305) The current example in the paper seems more exploratory in nature, making it hard to assess the method correctly.

Minor comments

How does Scoary2 handle linkage disequilibrium? This is mentioned as a problem in the introduction but not addressed in the paper. Many of the (human) GWAS methods / linear mixed model based methods can be adapted to bacteria by including a LD pruning step and a population structure correction term in the model, which the introduction ignores.

One of the contributions in the paper is the rewrite of Scoary to support newer orthology inference software. Does the code rely on using OrthoFinder specifically, or is it easy to plug in results from other methods without another rewrite?

Although the authors have made Scoary2 available in a variety of ways, please additionally consider adding Scoary2 to (bio)conda if possible.

Does Scoary2 in general handle multi-strain/metagenomic data as presented in the benchmark, or is this limited to this particular benchmark? Is the number of strains in the input somehow modelled in Scoary2?

The authors claim that linear mixed models / LASSO (I'm assuming this refers to LASSO regularization in regression models?) are hard to understand. I feel this is a somewhat unfair statement to make considering that a major advantage of linear models is their interpretability and the decades of research into this.

For future research into microbial GWAS, the authors recommend use of realistic simulations, real datasets, and flesh out guidelines for recommending tools for different scenarios. It would be interesting to see these aspects considered for Scoary2, too, if possible/realistic for the scope of the paper.

**Authors Response**

**Point-by-point responses to the reviewers’ comments:**

Authors’ responses to reviewer #1 and #2

(i) Response to reviewer #1

The authors would like to thank reviewer #1 for the thorough review of the manuscript and the very useful comments, which helped us to improve the manuscript.

Below you will find a brief summary of the most important changes to the revised manuscript:

- Change of focus: We now emphasize the software improvements and greatly reduce our focus on fermented foods and yogurt metabolome. From our point of view, the most important enhancement compared to original Scoary is the addition of the data exploration app and the resulting ability to process large phenotypic datasets.

- We turned our data exploration app into a standalone software library that might be used for other mGWAS tools.

- Benchmarking (i) the automatic binarization (new Figure 1), (ii) pairwise comparisons (new Figure S1), and (iii) the runtime.

- Simplified Figure 5 (previously Figure 4)

- Scoary2 is now on Zenodo, and we added installation instructions for conda

Please note that the performance details have changed since the original draft. This is mainly due to a hardware update. For example, on the old laptop, the Scoary2 pairwise comparisons algorithm was 40x faster than that of original Scoary, but on the new laptop it’s only 30x faster.

Please find our reply on the individual points raised by reviewer #1 below.

*Reviewer #1 comment:*

*This manuscript is essentially a fusion of two quite distinct papers. The first describes updating the popular Scoary software for pangenome GWAS analysis originally published in 2016. The second is analysis of metabolites in experimental yoghurt cultures, which uses the new Scoary2 software, which serves as a proof of concept of using Scoary for high-dimensional analysis combining GWas and metabolite data.*

*Most changes in Scoary2 are "under the hood" software engineering enhancements that allow the program to run faster and handle larger data sets and new output formats.*

Authors’ reply:

Thank you for the helpful summary. We have substantially revised the manuscript to put the focus on the improvements to the original software. From our point of view, the most important enhancement compared to original Scoary is the addition of the data exploration app and the resulting ability to process large phenotypic datasets. Furthermore, we have developed the data exploration app into a standalone software library (github.com/MrTomRod/mgwas-data-exploration-app) which can now be used for other mGWAS tools, such as TreeWAS and PySEER, etc.

*Reviewer #1 comment:*

*Another new feature is the ability to binarize continuous variables using a couple of well-known approach. These updates will certainly be welcomed. While the original software was popular for bacterial GWAS, it slowed down considerably when run on more than a few hundred genomes. Nevertheless, the new software is still limited to 13,000 input isolates, which is small compared the current frenetic pace of bacterial genomes sequencing (some species already have 100,000s of public genomes) and limits future its use truly large scale phenotypic testing.*

Authors’ reply:

Indeed, we experimented with stack-based implementation of the tree parsing algorithm which would remove the recursion depth limitation. This code still exists (https://t.ly/segPI), and should this need arise, we could switch to it. However, we decided against using it because it is slower, and our goal was to optimize for many phenotypes rather than many isolates. Moreover, if such a large dataset were available, a different mGWAS algorithm may be more suitable, for example one that is able to model gene copy numbers, detect interactions or weak effects to take full advantage of the increased power.

*Reviewer #1 comment:*

*The software was run on yogurts made using 44 different strains of Propionibacterium freudenreichii with a species pangenome of 1,466 hierarchical orthogroups. Thousands of traits - compounds identified by LC-MS were generated for each sample. Exploring the output of SCOARY analysis revealed a cluster of carnitine compounds were correlated in their concentration and were associated two previously unannotated genes near the carnitine biosynthesis cluster. The functions of these genes were not validated experimentally.*

*This combination is awkward, for example the two paragraphs of the intro doesn't mention the Scoary software, which is advertised by the title as the main theme but instead discusses the genomics of food fermentation. In general, the article spends more time on the results of the yoghurt metabolite study rather than the software. The discussion in particular is over-long and extrapolates the results from the proof-of-concept study without the benefit of any experimental follow-up. Each of the sub-themes is an iterative rather, than highly significant advance on the previous state of the art.*

Authors’ reply:

Thank you for this valuable comment. We agree with this assessment. Accordingly, we have significantly changed our manuscript, emphasizing the software improvements (especially the data exploration app) and the resulting ability to process large phenotypic datasets. We have greatly reduced our focus on fermented foods and yogurt metabolome.

*Reviewer #1 comment (Page 5-6 "Scoary2 results" section)*

*It is not clear what the 3889 traits measured are - that number is not mentioned in the methods. The section mentions "1,256 metabolites". How are these related to the 3,889 traits? Clarifications needed in this section.*

Authors’ reply:

Thank you for this helpful comment. The 3889 traits were filtered. We have clarified this point in the text with the following sentence: “(…) traits without at least one gene with a significant Fisher’s q-value are automatically removed from the output”.

*Reviewer #1 comment:*

*Also - were the p-values corrected for multiple hypothesis tests (i.e., from screening 3,889 traits).*

Authors’ reply:

Thank you for this very important comment, as previously, Scoary2 did not properly adjust the p-values for the many traits tested. We updated the algorithm, so that this is now the default behavior, that can be deactivated with the flag “--trait-wise-correction”.

*Reviewer #1 comment:*

*Page 5 line 55*

*There doesn't seem to be a point to introducing the first dataset that wasn't analyzed in this paper. It illustrates software performance but only anecdotally.*

Authors’ reply:

Thank you for this helpful comment. The full metabolomics dataset of 182 yoghurts is presented to illustrate the population structure challenge on an actual dataset and to emphasize the need for mGWAS methods. We have revised the text to highlight this point more clearly.

*Reviewer #1 comment:*

*A detailed study of performance under different parameters is missing from the study.*

Authors’ reply:

Thank you for this remark. To showcase the performance of Scoary2, we have now added three benchmarks, each with a slightly different focus:

Benchmarking the pairwise comparisons algorithm is the most time-consuming step, which depends on the number of isolates and genes. We analyzed our benchmark and determined the algorithm’s time complexity as well as the performance improvement. The results are available in the new Figure S1 of the revised manuscript.

Benchmarking the runtime of Scoary and Scoary2 on 100 randomly selected traits shows the performance improvement of the full pipeline on a real dataset.

Benchmarking the automatic binarization illustrates how strong the effect size of a gene must be to be picked up by the Scoary2 pipeline. The results are available in the new Figure 1 of the revised manuscript.

*Reviewer #1 comment (Page 6 line 5)*

*I found this para a bit confusing because it talks about clustering of traits between isolates and not GWAS. This could be made clearer.*

Authors’ reply:

Thank you for the helpful comment. We have revised the paragraph for clarification and now use consistently the terms "groups of metabolites" and "gene clusters".

*Reviewer #1 comment:*

*The figures (especially figure 4) contain too much information and make it hard to parse out the key results.*

Authors’ reply:

Thank you for this observation. Figure 5 (previously Figure 4) indeed contains a lot of information from different domains, which is in fact the point of Scoary2: it enables data integration of many phenotypes and genomes (mGWAS), but also includes phenotype metadata, gene annotations and takes genetic neighborhood into account (if connected to OpenGenomeBrowser). All of this was required to identify and begin to understand the significance of the carnitine-related metabolites and genes.

However, we revised and simplified Figure 5 as much as possible to make the main message easier to understand at first sight.

*Reviewer #1 comment:*

*Screenshots of software output should be in the supplement for a research article.*

Authors’ reply:

Thank you for this valuable comment. Since we decided to focus on the software side of our work, the data exploration app seems to us to be the key development that enables data integration and the analysis of large phenotypic datasets. Especially since we have further increased the utility of the data exploration app by developing it into a standalone software library which could be re-used for other mGWAS tools, such as TreeWAS, PySEER, etc. Therefore, we have decided to keep the screenshots of the data exploration app in the main text.

(ii) Response to reviewer #2

The authors would like to thank reviewer #2 for their valuable comments, which helped us a lot to rethink the presentation of our results and to improve our manuscript.

Below you will find a brief summary of the most important changes to the revised manuscript:

- Change of focus: We now emphasize the software improvements and greatly reduce our focus on fermented foods and yogurt metabolome. From our point of view, the most important enhancement compared to original Scoary is the addition of the data exploration app and the resulting ability to process large phenotypic datasets.

- We turned our data exploration app into a standalone software library that might be used for other mGWAS tools.

- Benchmarking (i) the automatic binarization (new Figure 1), (ii) pairwise comparisons (new Figure S1), and (iii) the runtime.

- Simplified Figure 5 (previously Figure 4)

- Scoary2 is now on Zenodo, and we added installation instructions for conda

Please note that the performance details have changed since the original draft. This is mainly due to a hardware update. For example, on the old laptop, the Scoary2 pairwise comparisons algorithm was 40x faster than that of original Scoary, but on the new laptop it’s only 30x faster.

Please find our reply on the individual points raised by reviewer #2 below.

*Reviewer #2:*

*The authors present Scoary2 - a rewrite of the Scoary method for performing microbial GWAS. In addition to rewriting Scoary, the v2 method adds an option to automatically binarize non-binary phenotype data with the use of a Gaussian mixture model or k-means clustering, support for newer orthology inference software, and a data exploration program. The features in Scoary2 are demonstrated on a single dataset consisting of multi-strain yogurt samples.*

*Since Scoary is undoubtedly widely used for microbial GWAS, a rewrite of the method will no doubt be well received and the data exploration subprogram in particular seems extremely useful to me. If the exploration program can be expanded to support other methods or supports a format that is easy to extract from other methods, then this would be a very useful development and a major new contribution in the paper. Regarding the new features in Scoary2, however, I think the paper needs some improvement in demonstrating their usefulness which I've outlined in more detail below.*

Authors’ reply:

Many thanks for this valuable suggestion. We absolutely agree on the usefulness of a universal data exploration app and consider it to be the main innovation of this work. Therefore, we decided to develop it into a standalone software library (github.com/MrTomRod/mgwas-data-exploration-app) which can now be used for other mGWAS tools, such as TreeWAS, PySEER, etc. It can be directly installed as a Python package but also has command line interface for tools that are not based on Python.

*Reviewer #2 major issues:*

*Scoary2 provides an option to automatically binarize non-binary phenotypes by clustering the phenotype observations into two clusters. I feel that this sort of automation should be done very carefully and only after consideration by the user, and in general it would be better to explicitly model non-binary features rather than forcing continuous/multi-category data to a binary format. In most cases where binarization is useful, it is (in my opinion) unlikely that the data would easily admit to binary phenotypes found with a simple clustering step. If this feature is retained in Scoary2, I think the paper needs to include a simulation or other benchmark that evaluates the performance of the binarization in a case where the data does not lend itself well to binarization.*

Authors’ reply:

We agree that, if possible, binarization should be done carefully and manually. We now point out more clearly in our manuscript that this feature is intended for large phenotypic dataset where manual binarization is not feasible.

We also agree that it would be better to explicitly model non-binary features and mention this in Future directions. However, it is beyond the scope of this manuscript.

As suggested by you, we decided to benchmark the automatic binarization using simulated datasets. The results are now presented in Figure 1 of the revised manuscript. We added a paragraph in the methods section explaining the procedure and made the corresponding code available on GitHub.

*Reviewer #2 comment:*

*The authors claim that other approaches to microbial GWAS such as linear mixed models make "hard-to-verify assumptions about bacterial evolution". It would be useful to explicitly write out what these assumptions are and include a benchmark demonstrating how Scoary2 handles cases where these assumptions do not hold and the other models fail.*

Authors’ reply:

Thank you for drawing our attention to this remark. The statement is in fact an error due to a mix-up of the text. With 'hard-to-verify assumptions about bacterial evolution', we originally referred to models like PhyC (Farhat et al, 2013; doi: 10.1038/ng.2747 ), which reconstructs ancestral mutations at all branches of the tree, Pagel's method, (Pagel 1994; https://www.jstor.org/stable/49836) which estimates transition rates, and Bayesian methods (Wolc et al. 2022; doi 10.1186/s12711-022-00724-8), where priors must be estimated. Somehow, part of the original text was retained while deleting this information. We removed the incorrect sentence from the relevant section (Comparison with existing mGWAS approaches).

*Reviewer #2 comment:*

*The population structure correction using a permutation test, if I understand it correctly, seems to ignore structure in the data that arises from eg. sampling many isolates from the same outbreak/source, or strong lineage structure in the input genomes. In this case the labels should ideally be permuted in a way that considers whole blocks/lineages at a time rather than switching a single label, or otherwise takes the phylogenetic relationships into account.*

Authors’ reply:

Thank you for this interesting question. Population structure is accounted for by our test statistic, the pairwise comparisons algorithm, which counts the number of contrasting and supporting pairs. We agree that the permutation test ignores the data structure. However, we disagree that label permutations should account for lineage structure. Completely random permutations are exactly what guarantees that the actual result is being compared to a set of test statistics generated under a null model, i.e., assuming no relation between genotype and phenotype but with tree structure remaining the same. As long as we only consider a single dependent variable, these permutation tests are highly robust.

*Reviewer #2 comment:*

*Scoary2 uses a pairwise comparison algorithm, which presumably scales quadratically in the number of inputs meaning that the method would unlikely scale to tens or hundreds of thousands of isolates. Is there an upper limit to how many isolates Scoary2 can realistically handle, or is it rather limited by the Python recursion depth to 13 000? The presented benchmark only contains 182 isolates, which makes it hard to evaluate the method's scaling.*

Authors’ reply:

Thank you for this suggestion. We benchmarked the pairwise comparisons algorithm, which depends on the number of isolates and genes. Based on our measurements, we determined the algorithm’s time complexity: O(n_genes * n_isolates). The results are visualized in Figure S1 and the code is available on Scoary2’s GitHub (https://github.com/MrTomRod/scoary-2/tree/master/benchmarking/picking_performance).

As the algorithm does not scale quadratically, we think the recursion depth should be the upper limit for the number of isolates that can be analyzed. On a side note, we experimented with stack-based implementation of the tree parsing algorithm which would remove the recursion depth limitation. This code still exists (https://t.ly/segPI), and should this need arise, we could switch to it. However, we decided against using it because it is slower, and our goal was to optimize for many phenotypes rather than many isolates. Moreover, if such a large dataset were available, we would propose to use a different mGWAS algorithm, for example one that is able to detect interactions to take full advantage of the increased power.

*Reviewer #2 comment:*

*To place Scoary2 in context with other approaches to microbial GWAS, it would be useful to see Scoary2 applied on a dataset where the genotype-phenotype associations have been extensively studied. One such possibility would be applying Scoary2 to the penicillin resistance data used in this tutorial: https://pyseer.readthedocs.io/en/master/tutorial.html. In this case the genetic determinants are well known (see eg. this review for this particular case https://www.microbiologyresearch.org/content/journal/mgen/10.1099/mgen.0.000305) The current example in the paper seems more exploratory in nature, making it hard to assess the method correctly.*

Authors’ reply:

Thank you for this comment. Our work is focused on further developing Scoary (performance improvements, binarization, data exploration app) so that it can be applied to large phenotypic datasets, rather than on establishing the Scoary method itself. Therefore, we refer to the comparison of different mGWAS methods already shown for Scoary1 and limit ourselves to showing that the new application works on the phenotypic dataset we describe in our paper. However, it would indeed be interesting to show a in depth-comparison of the performance of different mGWAS methods with different published data sets. Unfortunately, such a comprehensive comparison is not possible within the scope of this work.

Nevertheless, we applied Scoary2 to the gene-count dataset, and the output can be found here (https://scoary.bioinformatics.unibe.ch/pyseer/trait.html?trait=penicillin). It correctly finds pbp1a. To find the other php-genes, it would be necessary to process the SNP dataset and create a Manhattan plot, for example using the Phandango data exploration app like in the PySEER tutorial, but we did not go this far.

*Reviewer #2 minor comments:*

*How does Scoary2 handle linkage disequilibrium? This is mentioned as a problem in the introduction but not addressed in the paper. Many of the (human) GWAS methods / linear mixed model based methods can be adapted to bacteria by including a LD pruning step and a population structure correction term in the model, which the introduction ignores.*

Authors’ reply:

We agree that the introduction did not provide a sufficiently broad overview of the available approaches. Since this is not the focus of our paper, we simplified this part and now merely refer to the review article by San et al. 2019 (doi: 10.3389/fmicb.2019.03119) .

The pairwise comparisons algorithm deals with linkage disequilibrium by focusing on evolutionary transitions as outlined in the original Scoary publication (Brynildsrud et al, 2016; doi: 10.1186/s13059-016-1108-8).

*Reviewer #2 comment:*

*One of the contributions in the paper is the rewrite of Scoary to support newer orthology inference software. Does the code rely on using OrthoFinder specifically, or is it easy to plug in results from other methods without another rewrite?*

Authors’ reply:

Thank you for this question. Two input formats are supported, see https://github.com/MrTomRod/scoary-2/wiki/Input#genes). The gene-count format is produced by Roary, the gene-list format by both Roary and OrthoFinder. The separator of the table can be specified as a parameter. In addition, Scoary2 retains the VCF parser script from original Scoary to convert this format into a format that is supported by Scoary2.

*Reviewer #2 comment:*

*Although the authors have made Scoary2 available in a variety of ways, please additionally consider adding Scoary2 to (bio)conda if possible.*

Authors’ reply:

Thank you for this suggestion. Whenever a new Scoary2 version is released on GitHub, a new Zenodo DOI will be generated automatically. Moreover, we added instructions (https://github.com/MrTomRod/scoary-2/wiki/Installation#conda) on how to install Scoary2 through conda on GitHub. However, we did not develop a dedicated conda package because Scoary2 can easily be added on top of a Python 3.10 conda environment with gxx.

*Reviewer #2 comment:*

*Does Scoary2 in general handle multi-strain/metagenomic data as presented in the benchmark, or is this limited to this particular benchmark? Is the number of strains in the input somehow modelled in Scoary2?*

Authors’ reply:

Thank you for this question. Since all yoghurts contain the same starter strains, we make the simplified assumption that the change in the metabolome of the yoghurts is caused by the genes of the added strain, either by mere metabolic ability or by interaction with the starter strains. However, at present, the Scoary2 algorithm cannot perform a multi-strain mGWAS that considers all genomes of the added strains individually.

*Reviewer #2 comment:*

*The authors claim that linear mixed models / LASSO (I'm assuming this refers to LASSO regularization in regression models?) are hard to understand. I feel this is a somewhat unfair statement to make considering that a major advantage of linear models is their interpretability and the decades of research into this.*

Authors’ reply:

Thank you for this comment. We agree with your statement. However, we would like to point out the problem that the model omits randomly relevant genes when selecting traits. For example, only one gene is selected from an operon with several identically correlated genes. This makes it difficult to test and compare the gene-metabolite associations calculated using different types of models. To make this point clearer, we have adapted the wording in the text. “While the parameters of LASSO models are easy to understand, LASSO may randomly choose one of multiple highly correlated genes and drop the others, ...”

*Reviewer #2 comment:*

*For future research into microbial GWAS, the authors recommend use of realistic simulations, real datasets, and flesh out guidelines for recommending tools for different scenarios. It would be interesting to see these aspects considered for Scoary2, too, if possible/realistic for the scope of the paper.*

Authors’ reply:

We agree that it would be interesting to consider a realistic simulation using real data sets also for Scoary2 to compare the software with other mGWAS methods. Unfortunately, it is not possible to conduct such a comparison within the scope of this paper.

**Second round of review**

**Reviewer 2**

The authors have incorporated some of my suggestions in their revision and (with a valid reason) ignored others, which has overall improved the manuscript. I think the revised manuscript better highlights and focuses on the strengths of the approach, and splitting the method into standalone data exploration app and the mGWAS method itself is a wise choice. I have no new suggestions or comments to make.
